# A qualitative study of anticipated barriers and facilitators to the implementation of nurse-delivered alcohol screening, brief intervention, and referral to treatment for hospitalized patients in a Veterans Affairs medical center

**DOI:** 10.1186/1940-0640-7-7

**Published:** 2012-05-02

**Authors:** Lauren Matukaitis Broyles, Keri L Rodriguez, Kevin L Kraemer, Mary Ann Sevick, Patrice A Price, Adam J Gordon

**Affiliations:** 1 Center for Health Equity Research and Promotion, Veterans Affairs (VA) Pittsburgh Healthcare System, 7180 Highland Drive, Bldg. 2, Rm. 4020W (151C-H), Pittsburgh, PA, 15206, USA; 2 Division of General Internal Medicine, Department of Medicine, School of Medicine, University of Pittsburgh, Pittsburgh, PA, USA; 3 Veterans Integrated Service Network 4 (VISN4) Mental Illness Research, Education, and Clinical Center, VA Pittsburgh Healthcare System, Pittsburgh, PA, USA; 4 Veterans Engineering Resource Center, VA Pittsburgh Healthcare System, Pittsburgh, PA, USA; 5 Center for Research on Health Care, University of Pittsburgh, Pittsburgh, PA, USA; 6 Critical Care Service Line, VA Pittsburgh Healthcare System, Pittsburgh, PA, USA

**Keywords:** Alcohol consumption, Alcoholism, Inpatients, Nursing, Nurses, Implementation, Screening, Counseling, Qualitative research, Focus groups

## Abstract

**Background:**

Unhealthy alcohol use includes the spectrum of alcohol consumption from risky drinking to alcohol use disorders. Routine alcohol screening, brief intervention (BI) and referral to treatment (RT) are commonly endorsed for improving the identification and management of unhealthy alcohol use in outpatient settings. However, factors which might impact screening, BI, and RT implementation in inpatient settings, particularly if delivered by nurses, are unknown, and must be identified to effectively plan randomized controlled trials (RCTs) of nurse-delivered BI. The purpose of this study was to identify the potential barriers and facilitators associated with nurse-delivered alcohol screening, BI and RT for hospitalized patients.

**Methods:**

We conducted audio-recorded focus groups with nurses from three medical-surgical units at a large urban Veterans Affairs Medical Center. Transcripts were analyzed using modified grounded theory techniques to identify key themes regarding anticipated barriers and facilitators to nurse-delivered screening, BI and RT in the inpatient setting.

**Results:**

A total of 33 medical-surgical nurses (97% female, 83% white) participated in one of seven focus groups. Nurses consistently anticipated the following barriers to nurse-delivered screening, BI, and RT for hospitalized patients: (1) lack of alcohol-related knowledge and skills; (2) limited interdisciplinary collaboration and communication around alcohol-related care; (3) inadequate alcohol assessment protocols and poor integration with the electronic medical record; (4) concerns about negative patient reaction and limited patient motivation to address alcohol use; (5) questionable compatibility of screening, BI and RT with the acute care paradigm and nursing role; and (6) logistical issues (e.g., lack of time/privacy). Suggested facilitators of nurse-delivered screening, BI, and RT focused on provider- and system-level factors related to: (1) improved provider knowledge, skills, communication, and collaboration; (2) expanded processes of care and nursing roles; and (3) enhanced electronic medical record features.

**Conclusions:**

RCTs of nurse-delivered alcohol BI for hospitalized patients should include consideration of the following elements: comprehensive provider education on alcohol screening, BI and RT; record-keeping systems which efficiently document and plan alcohol-related care; a hybrid model of implementation featuring active roles for interdisciplinary generalists and specialists; and ongoing partnerships to facilitate generation of additional evidence for BI efficacy in hospitalized patients.

## Background

Unhealthy alcohol use includes the spectrum of alcohol consumption ranging from risky drinking, defined as >14 standard drinks/week or >4/occasion for men, and >7 standard drinks/week or >3/occasion for women and healthy individuals age 65 or older, to *alcohol use disorders*, defined as alcohol abuse and alcohol dependence [1-3]. Unhealthy alcohol use contributes to substantial morbidity, mortality, and social problems, but often goes unrecognized and unaddressed by healthcare providers [4-6]. A set of clinical strategies referred to collectively as alcohol screening, brief intervention, and referral to treatment (SBIRT) is recommended for improving the identification and management of unhealthy alcohol use [2,7-10]. Screening determines the extent of alcohol use and identifies the appropriate level of intervention needed, if any. Brief Intervention (BI) is a non-confrontational, patient-centered approach to risky alcohol use which involves a five- to fifteen-minute semi-structured motivational discussion raising awareness of alcohol-related consequences and motivating a patient toward behavior change [7]. This personalized patient-provider discussion provides the patient with feedback on his/her alcohol use, individualizes the relevant alcohol-related risks, explores readiness to cut-down or quit altogether, and explores concrete self-selected strategies for doing so [7]. BI has been shown to significantly reduce alcohol consumption, morbidity, and healthcare utilization in primary care patients [11,12], and has demonstrated potential, but inconclusive efficacy for patients in emergency and trauma care settings [13-16]. Referral to Treatment (RT) provides those complex patients who need more extensive alcohol-related treatment with referral to specialty care (e.g., addiction medicine/psychiatry providers, detoxification services, outpatient counseling, and self-help groups) [7]. To date, the clinical practices that have been studied most extensively are screening and BI, and evidence in support of RT among patients whose unhealthy alcohol use is identified by population-based screening is lacking. As a result, screening and BI (as opposed to RT) are most widely recommended, and most previous implementation studies have focused on implementation of screening and BI only. Specifically, alcohol screening and BI is included in primary care clinical practice guidelines issued by the United States (U.S.) Preventive Services Task Force, the U.S. Department of Veterans Affairs/Department of Defense [2,8], practice statements issued by the American College of Obstetricians and Gynecologists [17], and trauma center accreditation standards issued by the American College of Surgeons [9].

Additionally, the U.S. Joint Commission recently released new hospital accreditation measures which include alcohol screening, BI, RT, pharmacotherapy, and follow-up for hospitalized patients [18,19]. Despite such aforementioned recommendations and mandates, uptake of screening, BI, and RT by healthcare providers in these settings is still relatively limited [20]. While several handbooks for their implementation have recently been released which serve as pragmatic planning guides for overcoming barriers to implementation and sustaining such programs [21,22], very little implementation guidance is available with respect to alcohol screening, BI, and RT in the inpatient care setting [23,24]. Reports of existing barriers to uptake tend to focus on provider-level barriers such as lack of alcohol-related knowledge, competing clinical priorities and lack of time, concerns about intrusiveness and damage to the patient-provider relationship, negative attitudes about substance users, and perceptions that alcohol screening, BI, and/or RT are not within one’s professional role or set of responsibilities [25-29]. However, a few studies have identified structural- or organizational- barriers to the implementation of screening, BI, and RT, including lack of: (1) integration into existing workflow, (2) managerial, administrative, or financial support, and (3) third party reimbursement for these services [29-31].

More active roles for nurses in the delivery of alcohol screening and BI have been proposed [32,33] and featured in descriptions of screening and BI implementation in acute care settings outside the U.S. [23,24] A model of nurse-delivered BI holds potential and appeal for U.S. hospitals as well because nurses have existing skills in health promotion and patient education, are the largest group of healthcare providers in U.S. hospitals, and have the greatest amount of extended patient contact. In addition to calls for new organizational models for the delivery of alcohol screening and BI, calls have been made for BI researchers to partner more effectively with front-line providers in order to understand the challenges and impediments they face in the actual provision of alcohol screening and BI services [34]. A primary goal of this partnership is to move beyond merely persuading providers to perform screening and BI, and to help shape the conditions that will facilitate its successful, meaningful use in clinical practice [34].

To fully substantiate and support the implementation of alcohol BI in the inpatient setting, additional randomized controlled trials (RCTs) of BI efficacy in hospitalized patients are needed [35,36], with viable brief interventions delivered under conditions parallel to those found in typical healthcare practice and systems of care. Additionally, BI delivery models that involve other healthcare professionals, such as nurses, need to be developed and tested. The perspectives of front–line providers can inform the design and testing of brief interventions which are not only efficacious, but clinically feasible and well-received, thus serving as an early bridge between efficacy testing and potential implementation. Thus, to support the development and execution of an RCT of nurse-delivered BI, a greater understanding of the potential barriers and facilitating factors for the delivery of alcohol BI by nurses in the inpatient setting is needed. Additionally, because screening and RT are considered prerequisites or corollaries of BI, and because little is known about their implementation in inpatient settings, we also sought to identify facilitators and barriers to these related practices.

## Methods

### Human subjects protections

This study was approved by the Research and Development service and the Institutional Review Board of the VA Pittsburgh Healthcare System (VAPHS).

### Design and theory

This is a qualitative study which used focus group methodology and a modified grounded theory approach for the development of the interview guide and data analyses [37]. Our modified grounded theory techniques involved both deductive and inductive reasoning. Specifically, from a deductive standpoint, our investigation was initially framed as a “diagnostic analysis” designed to provide early consideration of factors which might enhance or impede the execution of a BI trial and potential BI implementation in the inpatient setting. Additionally, based on existing screening and BI implementation research, [28,30,31] and major implementation science theories [38], we made several assumptions. First, we assumed that potential facilitators and barriers to possible implementation existed [28,38] and second, that these barriers and facilitators might vary according to each component of SBIRT, i.e., screening, BI, and RT [31]. Consistent with these assumptions and previously identified barriers to the uptake and utilization of alcohol screening, BI, and RT [28,30,31], we developed our semi-structured interview guide to (1) assess current practices in alcohol-related care, and (2) prospectively identify the potential barriers and facilitators associated with the implementation of each SBIRT component, by nurses, in order to acquire in advance a determination of project feasibility [39]. From an inductive standpoint, all themes were derived from the data. Please see a detailed description of the inductive coding and analytic processes below.

### Participants and setting

Between February and April of 2010 we conducted seven 60-minute audio-recorded focus groups with 33 registered nurses who were providing direct patient care in full- or part-time staff nurse positions on three medical-surgical units (n = 135) at a large, urban university-affiliated VA medical center. VA/Department of Defense Clinical Practice Guidelines recommend annual alcohol screening (and BI/RT, if needed) for all patients in VA primary care settings [8,40]. Additionally, VA annual performance measures pertaining to alcohol screening and brief intervention have also been implemented for primary care settings [41,42]. While alcohol screening, BI, and RT, nurse-delivered or otherwise, is not standard practice for inpatient settings at this facility, a substance abuse consultation-liaison service is available for inpatient providers. This service, staffed by three addiction medicine physicians and one physician assistant, provides assessment of substance use, assistance with detoxification and withdrawal management, initiation or maintenance of pharmacotherapy for relapse prevention, and liaison to specialized addiction treatment. In addition, a full complement of (1) inpatient and outpatient, (2) acute and long-term, and (3) rehabilitation, after-care, and self-help addiction services are available to patients upon referral from inpatient and outpatient healthcare providers.

A comprehensive description and analysis of participant selection and recruitment in this study are contained in a previously published methodological article [43]. Briefly, nurses were recruited via email and information sessions held on the units at shift change [43]. Because patient clinical characteristics, professional culture, and workflow patterns differ substantially across care settings, as do the specific patient care responsibilities among different types of healthcare providers [30], we anticipated that potential barriers and facilitators to nurse-delivered screening, BI, and RT might be unit-specific. Subsequently, focus groups were comprised of nurses from the same unit, though nurses could be from day, night, or evening shifts. Due to federal ethics regulations prohibiting remuneration for research participation occurring during official work time [44], focus groups were conducted during participating nurses’ off-duty hours.

### Data collection procedures

Focus groups were conducted in an easily accessible, private conference room. Focus groups were moderated by a study Co-I (KLR) who is a female, doctorally-trained medical sociologist/VA health services researcher with extensive experience in qualitative focus group script development, facilitation, and analysis techniques. Aside from having assisted with recruitment, the moderator was unknown to the participants. The study coordinator was also present during each focus group session in order to conduct initial written informed consent procedures, take written field notes of observable group behavior to supplement the audio-recordings, operate audio-visual equipment, and distribute demographic surveys and participant remuneration; however, it is important to note that the study coordinator sat in the far corner of the room and did not participate in focus group discussion. A bachelor’s-prepared family nurse practitioner student (PAP) who is a critical care nurse at the VA facility was also present at three of the focus group sessions. The student took written field notes but also sat in the far corner of the room and did not participate in focus group discussion.

At the onset of each focus group, the moderator identified her role as indicated above and those of the study staff, and disclosed her non-clinician status. Focus group discussion followed a semi-structured interview guide (Table 1) developed by the PI and the moderator Co-I. The script was further reviewed by an additional study Co-I (AJG), a primary care/addiction medicine physician and alcohol health services researcher, but was not pilot-tested with nurses. The focus groups began by asking participants about existing clinical practices for addressing patient unhealthy alcohol use and perceptions of the nursing role in alcohol screening and intervention. For the purposes of introducing the practices of alcohol screening, BI, and RT, the nurses then viewed a six-minute video of a nurse practitioner performing BI [45]. The focus group discussions then resumed, centering on general features of the (1) inpatient setting, (2) nurse-patient relationship, (3) individual inpatient units, and (4) the VA healthcare system in general which could impact delivery of each of the three individual components of nurse-delivered SBIRT. When needed, prompts were provided by the focus group moderator as indicated in the interview guide.

**Table 1:** Focus group interview guide

**Opening questions**	
(1)	Tell me about what you have experienced with respect to alcohol use in your patients.
	*Probes:*
	- What are the biggest issues and needs?
(2)	How is unhealthy alcohol use typically addressed on your units?
	*Probes:*
	- What *formal* protocols/procedures/pathways are currently in place? Do other *informal* processes/practices exist?
(3)	Who currently bears responsibility for addressing unhealthy alcohol use? What is nursing’s current role and set of responsibilities?
***Introducing the idea of alcohol screening, brief intervention (BI), and referral to treatment (RT)***	
Viewing of 6-minute BI demonstration video [36]	
(4)	Is there a role for this type of alcohol screening and intervention in the inpatient care setting?
	*Probes:*
	- What would it look like?
	- How could it be incorporated into the inpatient setting?
	--How might it need to be tailored/modified?
(5)	What do you see as the nurse’s role in alcohol screening, intervention, and referral?
***Facilitators & barriers to performing alcohol screening, BI, and RT in the inpatient care setting***	
(6)	What are some of the potential facilitators of nurses doing alcohol screening in the inpatient setting, i.e., features of the inpatient setting, the nurse-patient relationship, your particular unit, or the VA in general that lend themselves well to ***alcohol screening*** for hospital inpatients?
(7)	What are some of the major *barriers* facing nurses when it comes to doing ***alcohol screening*** in the inpatient setting?
(8)	Let’s think about the facilitators and barriers to the next dimension of care, that is, brief interventions. What are the *facilitators* of nurses conducting ***brief interventions*** in the inpatient setting?
(9)	And what are some of the major *barriers* facing nurses when it comes to potentially doing ***brief interventions*** in the inpatient setting?
	*Probes:*
	- Other providers in other settings have reported:
	Lack of knowledge, skills, training, experience
	Lack of time, resources
	Role responsibility issues (not my role/job)
	Lack of role support
	Lack of colleague, administrative, institutional, clerical support
	Potential privacy issues/threat to patient-provider relationship
	Don’t like these patients
(10)	Finally, are there certain *facilitators and barriers* to nurses *making referrals to treatment* in the inpatient setting?
***Concluding questions***	
(11)	Is there anything else that we didn’t talk about today that you think is important for us to know? Is there anything you would like to add?

After the focus group, nurses completed an anonymous, 28-item questionnaire assessing their basic demographic information alcohol related education, volume/frequency of caring for patients with alcohol issues, and existing alcohol-related care practices. For their focus group participation, each nurse received $50 in the form of coupon vouchers that could be used like cash at the facility’s canteen, retail store, or coffee kiosk. Meals were also provided during each focus group session. After each focus group session, audio-recordings and field notes were transcribed verbatim into Microsoft Word by the study coordinator for data coding.

### Data coding and analysis

Focus group transcript data were analyzed using the grounded theory technique of constant comparison [37]. In our analysis, we used the constant comparative method to compare newly gathered data with previously collected data in order to develop categories of responses [46]. All transcripts were separately dual-coded by both coders; the coding process commenced immediately after the first group and continued beyond focus group completion. Through initial review of each individual session transcript by the PI (LMB) and the Co-I moderator (KLR), open coding allowed for identification of basic concepts related to barriers and facilitators of screening, BI, and RT. A provisional code list was initially generated from these first-level codes; codes were then operationally defined, refined, and agreed upon by the two coders. The properties and dimensions of these codes and their subcategories were then further defined, such as in terms of their frequency and intensity. Similar or related codes were collapsed into focused codes in order to represent interrelationships, variations, and underlying patterns in the data [37], allowing for the identification of anticipated barriers and facilitators of nurse-delivered screening, BI, and RT in inpatient settings. The codebook was finalized after review of the first two transcripts; however, we remained receptive to potential new codes emergent in the five remaining transcripts. The study coding tree is presented in Table 2.

**Table 2:** **Coding tree for anticipated barriers and facilitators associated with nurse-delivered screening, BI**^**a**^**and RT**^**b**^

**BARRIERS**	
	Patient-level
	➢ Concerns about negative patient reaction and limited patient motivation to address alcohol use
	· Patient expressions of anger, denial, dishonesty, offense, aggression, disinterest in changing
	◾ Alcohol-dependent patients
	-- Challenging behavior
	-- Repeated admissions
	◾ Sex and age-related differentials between nurse and patient
	Provider-level
	➢ Lack of nurse training and skills in alcohol screening, BI, and RT
	· Alcohol-related knowledge
	◾ Conceptual definitions, clinical criteria, established standards/recommendations
	· Alcohol-related skills
	◾ Effective therapeutic communication techniques
	◾ Goal-setting for consumption reduction
	➢ Limited interdisciplinary collaboration and communication around alcohol-related care
	· Differences in prioritization and attention to alcohol issue across provider disciplines
	◾ Physician resistance/reluctance to address alcohol use or withdrawal
	· Lack of effective communication with physicians, specialists
	· Lack of shared care planning with physicians, specialists
	➢ Questionable compatibility of alcohol screening, BI, and RT with the nursing role
	· Competing priorities, goals
	· Nursing advocacy and autonomy
	System-level
	➢ Inadequate alcohol assessment protocols and poor integration with the EMR^c^
	· Brevity of alcohol-related content in admission assessment
	· Despite admission template, lack of standardization in alcohol assessment across nurses
	· Limits of EMR regarding alcohol-related care planning
	◾ Lack of detailed patient care templates
	◾ Lack of guidance on follow-up actions
	◾ Inappropriately-generated automatic prompts for consults
	➢ Questionable compatibility of screening, BI, and RT with the acute care paradigm
	◾ Competing priorities, goals
	➢ Logistical issues
	· Lack of time
	◾ Task prioritization
	◾ Uninterrupted time
	· Lack of patient privacy
**FACILITATORS**	
	Patient-level
	· N/A
	Provider-level
	➢ Improved provider knowledge, skills, communication, and collaboration
	· Alcohol and screening, BI, RT education for nurses and doctors
	◾ General knowledge, brief intervention skills, communication techniques
	· Shared assessment, care planning, sense of responsibility
	◾ Inclusion of all disciplines’ professional perspectives
	System-level
	➢ Enhanced EMR features for alcohol-related care
	· Electronic templates and scoring for patient screening, assessment
	· Clinical decision making algorithms/electronic reminders
	· Consultation orders linked to assessment
	· Patient education resources
	➢ Expanded processes of care and nursing roles
	· Autonomy to initiate addiction specialist consultations
	· Specialized nurse educators/specialist team focused on BI and patient education

Notes: ^a^ BI = brief intervention; ^b^ RT = referral to treatment; ^c^ EMR = electronic medical record.

During weekly meetings throughout the coding process, the PI and Co-PI discussed emergent analytic themes and when needed, negotiated interpretive consensus. No changes were made to the interview guide during this process. We concurred that theoretical saturation, the point at which no new properties, dimensions, or relationships emerged from our data [37], was reached after the fifth focus group (representing nurses from three units), however, we conducted two additional focus groups to ensure that saturation had indeed occurred.

Field notes generated by the study coordinator and nurse practitioner student during the focus group session were reviewed by the PI for additional contextual information to inform interpretation of the data, including gestures and body language and participant seating arrangements. Because we employed a variety of widely used strategies for establishing the credibility and trustworthiness of our interpretations, we did not perform member checking, i.e., asking study participants to check or “approve” particular aspects of the interpretation of the data they provided. Data quality was assessed through a) checking for researcher effects (field notes taken by one of two observers for each focus group, peer debriefing by co-authors from nursing and medicine), and b) triangulating researchers (LMB and KLR from two different professional disciplines) [47,48]. We also engaged in extensive peer debriefing with co-author PAP, a critical care staff nurse at the same facility, to confirm interpretations and provide an external check on the inquiry process [48]. Furthermore, our interpretive flexibility is reflected in our presentation of negative evidence and rival explanations [47]. Finally, we used descriptive statistics to characterize the sociodemographic and other basic characteristics of the participants.

## Results

### Participant characteristics

In total, 33 nurses participated in seven focus groups, with four to eight nurses participating in each group (mean per group = 4.8). Participating nurses were predominantly female (97%), white (83%), and less than 50 years of age (85%). Additional sociodemographic, education- and practice-related characteristics are presented in Table 3.

**Table 3:** Characteristics of medical-surgical nurses participating in focus groups

**Characteristic**	**n (%)**^**a**^
**Sex**	
Female	32(97)
Male	1(3)
**Age Group**	
18-30 years	8(24)
31-40 years	10(30)
41-50 years	10(30)
51-60 years	2(6)
61-70 years	3(9)
**Race**	
American Indian/Alaska Native	1(3)
Asian	1(3)
Black or African American	5(15)
Native Hawaiian/Pacific Islander	0(0)
White	26(83)
More than One Race	0(0)
**Highest Education in Nursing**	
Diploma	7(21)
Associate	12(36)
Bachelor	12(36)
Master	2(6)
**Years as an RN**^**b**^	
Less than 1 year	0(0)
1-5 years	20(61)
6-10 years	3(9)
More than 10 years	10(30)
**Years as an RN with VA**^**c**^	
Less than 1 year	2(6)
1-5 years	22(67)
6-10 years	3(9)
More than 10 years	6(18)
**Estimated Hours of Alcohol-related Content Received in Nursing Education**	
None	1(3)
1-4 hours	12(30)
5-10 hours	7(18)
11-15 hours	3(8)
More than 15 hours	4(10)
Don’t know/Can’t recall	6(15)
**Receipt of Any Alcohol-related Continuing Education?**^**d**^	
Yes	17(52)
No	16(49)

^a^ Numbers may not total 100% due to rounding. No missing data.

^b^ RN = Registered Nurse.

^c^ VA = Veterans Affairs.

^d^ Types of alcohol-related education included: lecture/workshop/seminar, conference, journal club, grand rounds, advanced certification, or other.

### Themes

In their discussions of anticipated barriers and facilitators associated with nurse-delivered alcohol screening, BI, and RT for hospitalized patients with unhealthy alcohol use, nurses consistently anticipated the following barriers: (1) nurses’ lack of alcohol-related knowledge and skills; (2) limited interdisciplinary collaboration and communication around alcohol-related care; (3) inadequate alcohol assessment protocols and poor integration with the electronic medical record (EMR); (4) concerns about negative patient reaction and limited patient motivation to address alcohol use; (5) questionable compatibility of alcohol screening, BI, and RT with the acute care paradigm and nursing role; and (6) logistical issues (e.g., lack of time/privacy). Suggested facilitators of nurse-delivered alcohol screening, BI, and RT focused on provider- and system-level factors related to: (1) improved provider knowledge, skills, communication, and collaboration; (2) expanded processes of care and nursing roles; and (3) enhanced EMR features.

In our descriptions and analysis below, we provide direct quotations from a variety of nurses from all seven focus groups in order to illuminate these themes. Within each quotation containing more than one nurse, nurses are numbered to identify each speaker in the exchange. Supplementary quotations illustrating barrier and facilitator themes are provided in Tables 4 and Table 5, respectively.

**Table 4:** Anticipated barriers to implementation of nurse-delivered screening, brief intervention, and referral to treatment

**Anticipated barriers**	**Supplementary examples of quotations**^**a**^
1. Nurses’ Lack of a Alcohol-related Knowledge and Skills	“You know, I’m a pretty new nurse. I’m not real comfortable speaking to them about stuff like that (alcohol) yet. Just the whole being a nurse in an acute care setting, you know? Everything you have to do and all the responsibilities and all, it’s a lot of learning.”
	“. . . [the nurse practitioner in the video] made [the patient in the video] feel like she was listening to her and not just coming, “Ok, here’s the question, what’s the answer? Here’s the question, what’s the answer?” So, not all nurses have that ability to do that. . .”
	“…Some people have a bad taste in their mouth when it comes to dealing with somebody with an addiction. We all have a bad feeling about when, “Oh, he’s a drinker,” you know?” (multiple agreement) . . . So I think it needs to be somebody that’s more compassionate, that understands, that doesn’t have that stereotype.”
2. Limited Interdisciplinary Collaboration and Communication around Alcohol-related Care	You know, we can suggest [an addiction consultation] to some [doctors], but if I don’t know those doctors and I suggest [a consultation] just out of the blue, they’re not going to listen to me.
	Sometimes I feel I pass things on and they never get anywhere, you know? Three days later you’re still passing things on, it’s like, c’mon, you know?
	Calling the doctor and saying “Listen this guy’s abusing alcohol, this guy’s abusing marijuana” and they’re like, “Whatever”-
	Nurse 1: I don’t think that [the physicians] really look at our notes. . . They don’t read, they don’t have time to read-
	Nurse 2: And to be honest, too, I think with nurses, everybody looks to see if [the addiction consultation] has been done, and if it’s done, we just all move on.
3. Inadequate alcohol assessment protocols and poor integration with the EMR^b^	“It’s hard because, I don’t feel there is a enough structured assessment tool for any of it (alcohol). And I feel like it just gets bypassed, especially in that group (risky drinkers).
	“If they say [they don’t drink] and if they don’t show signs and symptoms (of alcohol withdrawal), it’s basically all just focused on what they’re there for.”
	“All that the admission assessment is requires, “How many drinks have you had? When was the last drink?” It’s not detailed- not really tell us or how to follow or make any commitment and all.”
4. Concerns about negative patient reactions and limited patient motivation to address alcohol use	Nurse 1: Sometimes the patients can be temperamental. You don’t want to cause a problem that’s not there, like, get them riled up.
	Nurse 2: And once get angry about one issue then they have trouble- they don’t want to take the meds for you, they don’t want to cooperate with anything else.
	Nurse 1: . . . (the older alcoholics), I think a lot of them are so far gone, you know?
	Nurse 2: That generation just doesn’t listen, especially to women.
	Nurse 3: Yeah, and I’ve had a lot of patients just tell me that “This is all I know, this is all I do.”
	Nurse 1: Our population is probably mid-50s to older--it’s something they’ve been doing for 25-30 years. . . at that point they don’t think they have a problem, it’s just normal to them.
	Nurse 2: Or it’s already too long. They’ve already got the problems that go with it (alcohol use), and think, why bother?
5. Questionable compatibility of alcohol screening, brief intervention, and referral to treatment with the acute care paradigm and nursing role	If they’re in for, like, something not alcohol-related, like pneumonia or whatever- -sometimes I think if the alcohol is not going to be an issue, as in they’re not going to withdrawal, it kind of gets overlooked and you just treat what’s medically wrong with them.
	. . .Sometimes it’s hard when they’re here for such a short period of time, to really get the big picture of what’s going on in their life, especially when a list of long medical problems that need to be addressed
	As far as acute care nursing goes, I don’t know what else we could do as nurses, other than what we do.
	And I hate to keep saying this but I really think that the people who are the professionals who are used to dealing with [alcohol] every day should be ones that are making goals with the patient.
	I actually think us as nurses, we do [alcohol-related counseling] automatically. I mean, we don’t need to be told, “Help your patients stop drinking.” We may not have all the necessary tools and it might be not the appropriate place but to get him over that acute phase of withdrawal, but to talk to him and try to encourage him to stop drinking, we do that all the time anyway.
6. Logistical Issues (e.g., lack of time/privacy)	I think that we’re so busy, and a lot of times we’re talking about the discharges, it’s like they’re handing you two admissions that are coming in and saying, you know, “Get your patient out of here, this guy’s coming in.” So to take a half an hour to talk to them about their drinking habits, like, it’s not gonna happen, you know?
	I don’t know if, as a nurse, on a typical day, I’d have that amount of time to sit with a patient--to build up a rapport back and forth (to discuss alcohol).
	Especially in a semi-private room. Who wants to talk about the most personal things in their life with, you know, some complete stranger next to them?

^a^ Quotations extracted from transcripts of 7 focus groups with 33 nurses from 3 medical-surgical units.

^b^ EMR = Electronic healthcare record.

**Table 5:** Anticipated facilitators to implementation of nurse-delivered screening, brief intervention, and referral to treatment

**Suggested facilitators**	**Supplementary examples of quotation**s^a^
1. Improved provider knowledge, skills, communication, and collaboration	“[We need] education on different communication techniques…resources we can teach patients about, and referral to treatment.”
	“. . . we could actually have someone give us a sheet that says something like “Here are some little pointers or tips on how to address these issues with your patient” because, like I said, I’ve been here my whole entire nursing career and not once have I ever had anybody tell me (that type of information).”
	“I think it should be nurses and doctors together. . . Both of our responsibilities- the whole idea of having two eyes see the same thing. We’re both asking them questions about alcohol, but yet nothing still is being done about [the patient’s alcohol use].”
	“if it’s all in [a shared] care plan, maybe it would be easier to address it and fit it in. . . when you’re going in to take care of the patient, you know, “Oh, I see you spoke to [the addiction specialists]-- how’s that going for you?”
	“. . .it’s advocating more for the patient . . more collaborative treatments with the physicians and being proactive about alcohol in our setting.”
2. Enhanced EMR^b^ features	Nurse 1: Maybe [the EMR] could just pop up whenever you’re doing the assessment and just put the (addiction) consult in.
	Nurse 2: Yeah, ‘cause I like that idea, that triangle (drinkers pyramid) diagram that you keep showing. On admission assessment, like-
	Nurse 3: Maybe if a patient falls in this section-
	Nurse 2: Yeah, falls in the top two tiers they need a consult and would it automatically pop up.
	“Something in the EMR like [the online patient education company]. I like it because you can give it to the patient, you give them the option, ““Would you like me to stay in here and discuss this with you, or would you like to have this and read over it?”
3. Expanded processes of care and nursing roles	Nurse 1: “I think [brief intervention] would be more effective if the patient had someone to be accountable to after discharge, also. ‘Cause they’re going to forget about us in three days but if they have that one steady person I think they’d be more likely to follow through.
	Nurse 2: Yeah, if you had, like, one special team that went around and did that,
	I think that’d be more beneficial.
	“[A facilitator would be] being able to make our own consults – put in our own consults – because maybe it’s being overlooked by someone else and we made a nursing decision thinking that based on what the patient’s telling us we can make our own consult to the [addiction consultation-liaison] team. Or we contact them directly . . . to advocate for that patient if we felt that they needed it.”
	“I think “the readiness ruler” (shown in video) is a very good tool that someone could use if we had maybe educators that came to the floor to take care of the patient, educate them one-on-one, like the diabetic educator that comes to the units.”

^a^ Quotations extracted from transcripts of 7 focus groups with 33 nurses from 3 medical-surgical units.

^b^ EMR = Electronic medical record.

### Anticipated barriers

#### Nurses’ lack of alcohol-related knowledge and skills

Inpatient nurses overwhelmingly cited their lack of alcohol-related knowledge and skills as a potential barrier to nurse-delivered screening, BI, and RT for hospitalized patients, particularly knowledge and skills outside the realm of managing acute alcohol withdrawal syndrome in patients with physical alcohol dependence. Many nurses reported a general inability to identify and classify alcohol risk, and an inability to define the appropriate goal or intervention for each level of risk.

For example, with respect to screening, one participant stated: “Everyone has a different opinion of what constitutes an alcoholic…or even what constitutes a problem.” Regarding BI, another nurse stated: “I’m not sure I know enough about what would be reasonable [drinking] goals for a patient. I don’t think I have enough training on that.” Nurses also specifically cited a lack of communication skills and a therapeutic style for effectively talking with patients about alcohol use.

Across focus groups, nurses were consistently unfamiliar with the quantities of alcohol that comprise a “standard drink” and unfamiliar with the recommended limits for low-risk drinking established by the National Institute on Alcohol Abuse and Alcoholism (NIAAA) [1]. Instead, nurses often described how personal benchmarking [49] about their own alcohol consumption patterns impacted their comfort (or lack thereof) and decisions around whether a patient was drinking to excess and/or in need of intervention. Below are several exemplary quotations from nurses.

. . . if they just tell me “Oh, I drink two beers a night.” Alright. I don’t see that as a problem, personally (group laughter). So, I’m not going to be like “Oh, this person’s an alcoholic, oh my gosh.

. . . if I drink when I’m off work, it’s going to be a barrier for me to really be talking to someone who’s trying to quit drinking. . .?

. . . I would feel like I was being hypocritical. I mean, who am I, you know . . . ?

#### Limited interdisciplinary collaboration and communication around alcohol-related care

Across focus groups, nurses also consistently mentioned incongruent degrees of attention and importance to alcohol issues across professional disciplines in healthcare. With regard to differences in the prioritization of addressing alcohol in the hospital, nurses noted the following:

Physicians treat you like you’re opening up a can of worms, like, this is going to be the reason that this guy is not going to be discharged, because you’re creating an issue out of this, or they need to be seen by [the addiction specialists] before they go…Or they’re like, ‘This doesn’t have anything to do with anything.’

. . . we had to really fight to get [a patient] the help that he needed and it was like pulling teeth trying to get him help . . . from the social workers, and, the doctors.

Another nurse described her frustration in attempting to communicate the need for more effective alcohol withdrawal prophylaxis:

Ninety-eight percent of the alcoholics are going need [medication-assisted withdrawal]. I just feel like [physicians] are not aggressive enough with the medication. And it’s not until, I think, the vitals start getting into it that they start listening. If we just say, “Hey, his eyes are very sensitive to light, he’s very nauseous, he has a massive headache, he’s very nervous, he’s got the tremors,” the physicians will say, “Oh, yeah, well, we knew he drank,” but once you throw the vital signs in there, then they start listening a little bit, but before that . . .

A few nurses in another focus group mentioned the lack of effective communication and shared care planning around alcohol-related care, as noted in the following exchange:

**Nurse 1**: [the addiction specialists] just kind of mosey in, and,

**Nurse 2**: And every time you place a consult, they’re like, “Thanks.”

**Nurse 3**: They don’t talk to us.

#### Inadequate alcohol assessment protocols and integration with the EMR

Nurses spoke extensively about the minimal alcohol-related assessment in the EMR, which prompts a few alcohol-related questions only upon admission, with little direction on how to use this screening-related information or to whom it should be communicated. In one exchange, nurses described the alcohol-related inpatient admission assessment in the EMR in the following way:

**Nurse 1**: We do the same assessment for every level [of drinking] (multiple agreement from participants). It doesn’t change, you know? (multiple agreement). I mean, somebody could say “Yeah, I drink but I have three, four beers a week.” Are they moderate, low-risk? You can’t identify it because it’s all the same question.

**Nurse 2**: [the EMR prompts you to ask] “How often do you drink, and for how many years?” and that’s it, point blank.

**Nurse 1**: Mmm hmm.

**Nurse 3**: And, “When did you have your last drink?” That’s- that’s it. (Multiple agreement).

Despite the intended standardization of assessment questions in the EMR, in another focus group, one nurse commented on the notion of alcohol BI by stating, “I don’t think there’s actually a standard [way of addressing alcohol], it’s just kind of on a whim. . . it depends on the nurse.”

In addition to discussing a lack of adequate EMR-based clinical decision aids for using and applying this screening information, nurses specifically discussed the lack of alcohol-related care plans in the EMR for responding to or intervening with the entire spectrum of drinkers, i.e., abstainers, low-risk drinkers, risky drinkers, and drinkers with alcohol use disorders. The following exchange illustrates nurses’ perceptions of this lack of consolidated, useful templates in the EMR for planning, documenting, and following up on alcohol-related care:

**Nurse 1**: There’s no [individual plan of care] template for alcohol…People are usually just clicking around in the notes.

**Nurse 2**: Click and send it out in space. And then sign and nothing really happens with them.

Finally, across focus groups, several nurses described how responding honestly to the current, dichotomously-worded alcohol screening questions in the EMR often generates prompts for substance abuse consultations that may not be appropriate:

On that admission assessment, let’s just say you occasionally drink, you know at a holiday or something like that. Technically, you should put “Yes,” they’re a drinker but . . . you may have an 85-year-old man who, his wife is right there and they’re all backing up that story saying, “He doesn’t drink,” but if you say “He has an occasional drink,” technically it generates the need for [an addiction specialist consultation]. . . So, where is the line?

#### Concerns about negative patient reactions and limited motivation to address alcohol use

With respect to alcohol screening and BI, nurses expressed concerns about patient denial, anger, offense, dishonesty, and even aggression in the context of alcohol-related discussions. For example, with respect to perceived patient denial, one nurse commented: “[They say] ‘I drink five beers a day, it’s not a big deal’ . . . they just don’t see that it’s an issue.” Regarding a lack of patient honesty, another nurse stated: “. . . they’re not always honest about how much they’re drinking.” In other focus groups, nurses described patient reactions to discussing alcohol in the following ways: “They get defensive,” “They say we’re lecturing them,” and “It definitely gets patients aggravated when you try and talk about things that they don’t think is related to why they’re there (multiple agreement from other nurses within the focus group).” One nurse described aggression from patients in the context of her previous alcohol-related discussions with patients during their hospital stays:

You try to go and say “Let’s discuss this,” and they will swear at you and throw stuff at you, and tell you “get out” . . . And the next time you come back to take care of a medical thing, they’re spitting at you, because they said, “I told you I don’t ever want to see you again.”

In all seven focus groups, nurses’ descriptions of negative patient reactions to screening and intervention attempts included references to extremely challenging patient behavior and lack of motivation to change exhibited by alcohol-dependent patients. Specifically, nurses made references to patients who were admitted frequently for alcohol detoxification or who went through alcohol withdrawal often during consecutive medically-related admissions. Nurses’ descriptions of working with these patients reflect the cyclical nature of addiction and relapse, as well as significant frustration over perceived failed attempts to refer patients to alcohol treatment:

They come in for the detox and once they get past the hard part and the [addiction consultation-liaison service] comes and sees them, then they’ll deny that they need help.”

So many of them come back so often with the same problem. It’s a revolving door. [They say] “I’m ready,” then hop on the transfer to [the substance abuse treatment facility], but then 6–8 months later they’re back doing the same thing.

I have seen “repeat offenders” coming in, um, and saying they want to go to rehab and they’ll come in, do the [Clinical Institute Withdrawal Assessment][50], get the medication, then they change their minds, but they’re coming right back in again [for detoxification again].

In other focus groups, however, nurses felt that the nurse-patient rapport developed after several admissions would actually facilitate BI and motivational counseling. Nurses explained that such rapport reduced the time needed to establish trust, promoted communication about alcohol, and helped patients feel understood and supported. One nurse explained:

This might be hard, but say you come in, and I’m your nurse and I talk with you about your problems with alcohol and we know each other . . . and the next time you come in hopefully I’m here again and I say, “Remember last time we were in this predicament?” I think on a personal level they would do better.

Another nurse described a positive encounter with an alcohol dependent patient in similar terms:

I said, “You’re back. What happened?” and he was like, “Yeah, yeah, I can’t do it, I couldn’t do it or something, but I’m gonna try it again.” I said, “Good, I’m glad you’re here. As long you know that we’re here for you, we’ll get you through” and he was so appreciative . . . that he’s here, and we came, and we said, “Ok, we’ll carry you through this again and get you out there and, you know, this will be better this time around.”

In some instances, nurses’ concerns about negative patient reactions to discussing alcohol use was associated with the second minor theme of perceived nurse-patient age-and sex-related differences; many of the nurses were younger and female, while the majority of Veteran patients tend to be older and male [51]. For example, in one focus group, the following discussion ensued between the moderator and a nurse participant after viewing the six-minute video on BI:

**Moderator:** OK, so, do you think that there’s a role for this type of alcohol screening and intervention in the inpatient care setting?

**Nurse :** Yes, but not with our population (laughter).

**Moderator:** OK, and why is that?

**Nurse :** Oh, goodness. It’s mostly geriatric patients. . . most of them are like, 50 and up, and usually the ones who you see are so, set in their ways, you know, these guys, they may be like, 70, and they’re just like “What do I care? I don’t have anything to lose.”

An excerpt from another discussion between three nurses also reflects varying opinions about discomfort with discussing alcohol in the context of nurse-patient age and sex differentials:

**Nurse 1**: . . . I think it would be a lot easier with a 21-year-old college student saying “This is what you should be doing” rather than me, a 20-something-year-old female talking to a 60-year-old lifetime drinker (laughter), being like “You need to change.” I think that that’s a huge part of it . . .

**Nurse 2**: That’s a big block . . .

**Nurse 1**: . . . (laughter) I mean, it seems completely backwards for me to be taking that role.

**Nurse 3**: . . . I think it would be harder to talk to someone my age about this. . . you know, you go to college, you do your thing, and I feel like if I was talking to someone more on my age level it would be more difficult for me. . .and I think we’ve been seeing more younger people come in (for inpatient care at this VA facility).

#### Questionable compatibility of alcohol screening, BI, and RT with the acute care paradigm and nursing role

Nurses sometimes expressed uncertainty about professional rights and extent of responsibility for addressing alcohol use, both as a nurse and as an inpatient care provider, and this was intertwined with uncertainty about how to blend the health promotion focus of alcohol screening, BI, and RT with the implicit goals of the inpatient setting. For example, one nurse stated: “And we’re on an inpatient acute medical unit. Our job is to stabilize and address the pressing issue, and so this is an issue of ‘Where does this fall and whose responsibility is it?’” Another participant noted: “A lot of times . . . when you tell the doctors [about alcohol use], it’s not their priority…a lot of times they think ‘We’re not their PCPs, we’re not dealing with their chronic issues.’”

On the other hand, many nurses mentioned patient advocacy as a component of their professional role, but differences existed with regard to the specific tasks they envisioned for themselves and for physicians. In one focus group, a nurse commented, “We’re patient advocates. We need to be pushing the doctors to order what our patients need. We do it all the time with pain medication and everything else . . .Why wouldn’t we do it with alcohol screening?” In another focus group from a different unit, the following exchange occurred between a nurse and the focus group moderator:

**Nurse**: As an advocate I think we should be able to initiate a consult for [the addiction consultation-liaison service], definitely, but. . .

**Moderator**: And can you do that currently?

**Nurse**: No.

#### Logistical issues

Some nurses also expressed uncertainty about their ability to perform alcohol screening, BI, and RT given logistical concerns, such as lack of time and patient privacy. Regarding time management, one participant noted: “Nursing is all about prioritizing your tasks…screening definitely gets put on the back burner…Prioritizing, that’s what they teach you to do!” Nurses also raised concerns about having uninterrupted time to perform a BI-style discussion. Expressing doubts about her potential ability to integrate BI into a typical day on her unit, another nurse commented, “I have to get in there, get as much done as I can before the doctors come up because then you get a med student in there, or, you’ve got [physicians] rounding . . . time is definitely limited, between distractions of being a nurse and then doctors interrupting and taking precedence over you.”

With respect to lack of patient privacy due to shared patient rooms, another nurse exchange within one focus group went as follows:

**Nurse 1**: A lot of times they’re in a room with someone else.

**Nurse 2**: I think a lot of them would feel uncomfortable . . . knowing someone’s listening on the other side.

### Suggested facilitators

Suggested facilitators of nurse-delivered alcohol screening, BI, and RT focused on provider- and system-level factors. While equal time was allotted during the focus group discussions for exploration of barriers and facilitators, nurses identified fewer potential facilitators. Our questions about facilitators were hypothetical questions that asked participants to determine potential facilitators to nurse-delivered alcohol screening, BI, and RT in the inpatient setting. Nonetheless, nurses in all seven of the focus groups were actively engaged in generating specific ideas and pragmatic suggestions for how implementation of nurse-delivered alcohol screening, BI, and RT could be facilitated.

#### Improved provider knowledge, skills, communication, and collaboration

With respect to alcohol screening and BI, nurses articulated explicit needs for learning more about what constitutes a standard drink, what constitutes risky drinking versus an alcohol use disorder, what appropriate interventions are for each risk level, and a desire to learn specific communication techniques and strategies for discussing alcohol use with patients. As one nurse stated:

I really think it’s a lack of nursing education on the subject (of alcohol) as a whole. I can tell you that drinking causes the heart problems and causes issues with diabetes and all that stuff, but as far as more specific things go, I really am not comfortable with my knowledge. I would definitely want to study more about that before I’d be comfortable having that kind of a (BI) conversation.

Regarding BI, another nurse suggested, “[We need] a proper little script on to actually approach the topic where you’re not aggressive--so they don’t think that you’re maybe attacking them or, coming across like, “Well. How much do you drink?”

Nurses also articulated the perceived need for alcohol-related education for physicians as well, particularly in the context of an academic medical center that serves as a medical residency training site for the affiliated university. Regarding interdisciplinary practice, one nurse commented:

. . . the doctors here are so new, most of them, and they’re changing so much that they’re not addressing these alcohol situations adequately. They’re not getting taught (about alcohol) -- they need to be educated better, and every three or four weeks they’re moving on. I think it’s just important that we remember the population we’re dealing with . . . the VA is not like every other hospital. . .

Nurses also spoke about the need for improved, face-to-face communication and shared care planning with physicians, addiction specialists, and social workers, particularly when RT was indicated for patients with alcohol dependence. One nurse said, “[We need] to have the [consultants] come speak to us and say . . . ‘this is our plan for this specific patient.’” In another focus group, nurses described a particular provider on the addiction consultation-liaison service who they felt exemplified good communication and collaborative practice:

**Nurse 1**: I think she takes the time . . . she’s just enthusiastic about her job-

**Nurse 2**: And she seems sincere and sincerely cares about her patients and getting them better and, you know?

**Nurse 3**: She’s just an excellent [provider]. She’s just very personable. She listens from the nurse’s perspective--and the patient’s perspective.

**Nurse 2**: Yeah – she does have very good rapport with the nurses.

**Nurse 1**: And is very involved and asks our opinions and our ideas.

Finally, one participant summarized shared responsibility for addressing alcohol this way:

People need to think more that this is their responsibility. . . in the back of your mind, you go, “Well, social services is going to make sure things are taken care of” . . . It’s everyone’s responsibility to make sure that [alcohol] is being addressed instead of thinking that someone else is going to be taking care of it.

#### Expanded processes of care and nursing roles

Participating nurses also generated ideas about creating expanded or unique nursing roles for promoting comprehensiveness and continuity of care. Suggestions included a new, exclusive role for a dedicated nurse alcohol educator/coordinator, and the development of a special team of nurses to approach and intervene with risky drinkers, as is noted in the following exchange:

**Nurse 1**: If [the patient] says, ‘Yeah, I’d like to quit drinking,’ have a job for one nurse to go talk to them.

**Nurse 2**: Or have a team on the floor, specifically-trained, that could take it further.

Other nurses suggested alcohol risk reduction follow-up visits or telephone calls by a home care or inpatient “discharge nurse,” and commented on the importance of “being able to initiate our own [addiction specialist consultations] . . . or contact them directly” (for patients with known or suspected alcohol withdrawal).

#### Enhanced EMR features

Nurses generated a myriad of ideas for how the EMR could be enhanced to facilitate nurse-led screening, BI, and RT for hospitalized patients. Specifically, nurses envisioned enhanced EMR features such as (1) automated scoring of alcohol screening instruments with subsequent prompts for addiction consultation based on those scores, (2) preset links to clinical algorithms based on patient’s alcohol-related risk level, (3) places to document BI and patient responses to that intervention, and (4) prompts to revisit the alcohol issue again later during hospitalization. For example, as one nurse suggested: “[The EMR] should have a more structured assessment tool [with] all of the medications and interventions that you use for the patients.” Another participant stated: “If a patient never shows a sign of alcohol withdrawal, I don’t ever think about it again . . . [we need] something there to remind us to bring it up again.”

Nurses also suggested easily available electronic patient education materials to support alcohol-related education and risk reduction discussions with patients, and described existing educational protocols on which alcohol-related patient education could be based. As one nurse suggested, “It could be like the [congestive heart failure] education . . . the charge nurse would get a list of patients who need the [alcohol] education.”

## Discussion

Alcohol screening, BI, and RT is a set of clinical strategies for the identification and management of unhealthy alcohol use. To date, early attempts to implement alcohol screening and BI have mainly occurred in primary care and have involved substantial challenges [52,53]. Other similar, federally-funded programmatic initiatives have been carried out in emergency/trauma settings [14,54]. Engaging direct healthcare providers in discussions and partnerships early is imperative for effective and sustained implementation of alcohol screening and BI in these and other potential healthcare settings [34]. Inpatient nurses in our study anticipated numerous provider-, patient-, and system-level barriers to nurse-led implementation of alcohol screening, BI, and RT, but also proactively suggested a variety of provider- and system-level facilitators of their delivery as well. To our knowledge, this study is one of only two U.S.-based studies exploring the potential implementation of alcohol screening, BI, and RT by nurses in hospital settings [55], and the only study to explicitly and comprehensively explore the perspectives of front-line providers for the purposes of BI trial design.

### Novel findings: Barriers and facilitators to nurse-delivered alcohol screening, BI, and RT for hospitalized patients

Novel findings from our study include the barriers of (1) limited interdisciplinary collaboration and communication around alcohol-related care, (2) inadequate alcohol assessment protocols and integration with the EMR, and (3) questionable compatibility of alcohol screening, BI, and RT with the acute care paradigm. In turn, the corresponding facilitators of (4) improved provider communication and collaboration, (5) expanded processes of care and nursing roles, and (6) enhanced EMR features, are also relatively novel and have previously received little discussion in the extant literature.

Nurses in our study viewed improved interdisciplinary communication and collaboration and new roles for nurses as essential prerequisites for, and facilitators of, potential nurse-delivered alcohol screening, BI, and RT in the inpatient setting. Alcohol BI and RT have traditionally been conceived primarily as physician responsibilities, with the majority of BI-related trainings and initiatives in the U.S. focused on in-house generalist physician models of BI/RT delivery [33], that is, models where existing physicians within a practice or facility assume responsibility for BI and RT tasks [14]. In a study of BI and RT implementation by emergency department nurses, participants made general recommendations regarding the development of a specially-trained interdisciplinary team to implement BI and referral to more specialized treatment [55]. However, models of team-delivered screening, BI, and RT, and consideration of the explicit roles which could be assumed by various healthcare professionals are largely absent in the literature.

Nurses in our study also explicitly describe reluctance or resistance to addressing alcohol-related issues among their physician colleagues. Some nurses attributed this resistance to a lack of awareness or education, residency training schedules, pressure to discharge patients in a timely manner, and competing medical priorities in the acute care setting. In this latter vein, our findings regarding the questionable compatibility of alcohol screening, BI, and RT with the acute care paradigm have also been minimally discussed elsewhere [56]. Additionally, other authors have explored primary care providers’ perceptions of their responsibilities with respect to treatment versus prevention [29]. In an era of rising healthcare costs, prevention and low-cost interventions are both appealing and prudent, but their introduction into settings with more acute agendas may be especially difficult, particularly if it challenges the perceived healthcare mission of that setting and providers who are already overwhelmed with other clinical responsibilities.

The VA’s EMR system was designed to provide clinicians, managers, support staff, researchers, and others an integrated patient record system. Otherwise known as the Computerized Patient Recordkeeping System (CPRS), the VA’s EMR allows point-of-care management of patient care and allows efficient access to and use of patient information. This single interface displays allergies, lab results, medications, existing orders, consults, and other clinical data in a format conducive to clinical decision making. Other features of CPRS include processing and storage of provider orders, clinical notifications and reminders, and consultation ordering/tracking [57]. Nurses working in VA healthcare settings thus rely heavily on the EMR for planning and documenting patient care. When envisioning how to potentially incorporate alcohol screening, BI, and RT into a typical day on their units, nurses participating in our focus groups overwhelmingly foresaw a prominent and central role for the EMR.

Few authors from outside the VA have explored the role of the EMR in facilitating the implementation of alcohol screening, BI, and RT by considering how EMRs could be configured and adapted to support clinical decision making and the documentation of patient care [31,49]. In one study of screening and BI implementation in a non-VA emergency department, nurses provided similar recommendations to those provided by nurses participating in our focus groups, namely, a computerized alcohol screening instrument, and immediate plans of patient care generated according to risk level [55].

### Nuanced findings validating the literature

The remainder of our findings are consistent with previous reports in the nascent literature on barriers to the implementation of alcohol screening and BI; first, “personal benchmarking” for determination of patients’ risk [24,28,49,58] and concerns about negative patient reactions and limited motivation to address alcohol use [24,28,59,60]. In particular, we describe several nuances to these findings which have only minimally been reported elsewhere. Sporadic attention has been given to how healthcare providers’ own alcohol consumption might affect the type or extent of alcohol risk reduction discussions that they have with patients [24,28,49,58]. Our findings related to nurses’ feelings of potential hypocrisy and the implicit determination of alcohol consumption greater than their own as problematic echo similar findings reported in other qualitative studies conducted with nurses [24,28] and physicians [49]. Other authors have reported relationships between nurses’ own alcohol consumption and their degree of satisfaction and personal comfort, and ability in working with drinkers [58]. These phenomena suggest that providers’ own alcohol consumption may be an important but relatively under-considered barrier to their uptake of evidence-based communication practices around alcohol risk reduction.

We identified nurse discomfort with alcohol-related discussion across different nurse-patient age and sex permutations, which has previously received only minimal attention [24,61]. Furthermore, while other types of healthcare providers (e.g., physicians) have also expressed concerns about potential negative patient reactions [24,28,60,62], nurses in our study are among the few reporting explicitly aggressive patient reactions in response to initiation of alcohol-related discussions [28]. The aforementioned nurse-patient age-sex differentials, as well as patient perceptions of different healthcare providers’ authority (i.e., nurses versus physicians), may serve as contributing factors in these reported instances of aggression and may explain the absence of similar reports in studies of physicians.

Finally, our findings also validate similar results previously reported in the literature, including (c) provider lack of alcohol-related knowledge and skills [25,28,59,60] (d) questionable compatibility of alcohol screening, BI, and RT with the nursing role [24,27,28,63], and (e) logistical issues such as time lack of time and privacy [55,60], In turn, provider education and training on alcohol screening and intervention tends to focus on developing adequate knowledge and skills in these practices. The extent to which provider training addresses the intra- and inter-personal undercurrents of alcohol communication such as “personal benchmarking” and “age-sex” differentials is unclear. Failure to adequately and candidly address social nuances such as these may be an important limitation in current training and implementation strategies for screening and BI.

### Implications

The alcohol-related standards approved by the Joint Commission in July 2011 [18,19] generated considerable controversy during their pilot-testing and development, in part due to concerns about the lack of solid and consistent evidence for the efficacy of all three components of alcohol S, BI, and RT in hospitalized patients [35,36]. This evidence will be essential for healthcare provider and hospital administrator buy-in during implementation initiatives. Additionally, the results of our study indicate the need for consideration of the following issues during the design of future BI trials and implementation strategies.

#### (1) Comprehensive provider education on screening and BI

Our findings suggest that conducting an RCT of nurse-delivered, inpatient BI will require increasing nurses’ basic capacity to screen and intervene for unhealthy alcohol use within an interdisciplinary inpatient care team. In turn, comprehensive continuing education and skills training will be needed for staff nurses potentially participating in screening and/or BI delivery. Furthermore, successful execution of a nurse-delivered BI trial would potentially impact processes of patient care in which other healthcare professionals participate as members of a team (e.g., referral to specialty care or initiation of pharmacotherapy for acute withdrawal). At a minimum, ancillary information sessions about screening and BI, as well as RT, would likely be required for colleagues such as physicians, social workers, and case managers. Training should be interdisciplinary, tailored to the culture, issues, and patient populations found in the inpatient setting, and should consist of standard core elements for all healthcare professionals with additional segments adapted for the patient care roles and responsibilities of providers representing different disciplines.

In addition to specific alcohol-related knowledge (e.g., standard drink, risky drinking, and low-risk drinking limits) and screening/BI skills, our findings suggest that nurse education should explicitly and meaningfully address (a) role ambivalence around performing screening and BI, (b) the role of health promotion in the acute care setting, (c) providers’ own use of substances/personal benchmarking about alcohol-related risk, (d) misperceptions about alcohol consumption and risk in patient subgroups such as older adults and women, (e) inpatients’ acceptability for alcohol screening-, BI-, and RT-related care tasks, and (f) unique challenges which might arise in the context of alcohol screening, BI, and RT (e.g., patient aggression or age-, sex-, or culturally-related nuances of provider-patient communication about alcohol use).

Educational approaches commonly used to train large groups of providers, such didactic lectures and online training modules, may be of limited value in teaching the skills needed by nurses to effectively deliver alcohol screening and BI. Because alcohol BI involves semi-structured discussion and therapeutic communication techniques, one of the challenges for nurse training efforts will be incorporating “booster sessions” and opportunities for skills modeling, role playing practice, and feedback; single-session training workshops are unlikely to provide the role support needed to sustain practice change over time [64,65]. In particular, because formal alcohol screening and BI likely involve unfamiliar skill sets and unfamiliar professional roles for most inpatient nurses, reiteration of training concepts, ongoing role support, and intervention monitoring will be essential for assurance of intervention fidelity in the context of an RCT.

In the context of personal discomfort with addressing alcohol, nurses and physicians have also acknowledged their ambivalence and variable compliance with professional guidelines and policies for addressing substance use [49]. While the literature on patient acceptability for alcohol screening, BI, and RT is sparse, patients nonetheless generally report comfort with alcohol screening, brief counseling, and alcohol-related discussions with their healthcare providers [66,67], even in inpatient settings [24,68]. The implementation of provider-level practice changes which involve sensitive behaviors or topics (e.g., alcohol/tobacco use, depression, intimate partner violence), presents challenges that implementation of other practice changes do not (e.g., hand washing initiatives, new catheter or chest tube insertion processes). The frequency with which nurses expressed discomfort about alcohol-related counseling light of their own alcohol use also suggests that when dealing with sensitive behaviors or topics, traditional study recruitment strategies might be less effective if issues of provider cognitive dissonance, conscience, or moral distress are not candidly but sensitively addressed.

#### (2) Record-keeping systems which efficiently document and plan alcohol-related care

Appropriate, thorough, and easy documentation of alcohol-related care is essential for continuity of care across healthcare settings and providers, and will be increasingly important as more healthcare systems switch to EMR, as alcohol-related hospital performance measures are adopted [17,18], and as alcohol screening, BI, and RT services become increasingly reimbursable by insurers [69]. EMR documentation can ideally identify patients with alcohol misuse, identify the specific components of brief intervention counseling provided, distinguish between alcohol- and drug-related counseling, and prompt providers to perform alcohol screening and BI [42]. EMR features which streamline clinical decision making can help facilitate the execution of practical “next steps” once unhealthy alcohol use is identified. Additional features can support straightforward documentation of screening, BI, as well as RT. In the outpatient setting within the VA, clinical reminders and decision support tools have been incorporated into the EMR for both alcohol screening and follow-up [41,52,70]; application of similar configurations could be applied to EMR templates and processes for the inpatient setting. Given however, that the current clinical reminders are “passive,” this EMR feature may serve as only one component of a multi-faceted strategy to facilitate provider adherence to the alcohol-related care guidelines.

#### (3) A hybrid model of implementation

Three general models of implementation for alcohol screening and BI have been utilized by programs receiving federal funding for BI implementation initiatives in the U.S.: (a) “In-house generalist” models where existing clinicians perform services, (b) “In-house specialist” models where existing behavioral health or other specialists are appointed to conduct services, and (c) “Contracted specialist” models where outside behavioral health or other specialists are hired to conduct services [14]. Trials comparing these models have not been published.

We propose testing of hybrid models of nurse-delivered alcohol screening and BI which feature innovative, active roles for nurses. Hybrid models of delivery could carve out specific roles and responsibilities for in-house generalist nurses, while assigning other responsibilities to in-house specialists such as teams of specially-trained staff nurses available to perform BI when needed, or psychiatric nurse or addiction/psychiatry consultation-liaison services. These types of hybrid models could harness the power of the nurses-provider relationship during hospitalization, and are highly consistent with Institute of Medicine “key messages” regarding transformation of practice through re-conceptualized roles for nurses which allow them to practice to the full extent of their education and training [71].

### Limitations

The generalizability of our findings may be limited because of the use of a single VA site and the use of nonprobability sampling techniques. Our ability to assess the representativeness of our sample is limited because for the time period during which data were collected, demographic data on registered nurses are only available at the facility level, as opposed to the unit or service-line (i.e., medical/surgical) level specifically. Nonetheless, compared with RNs across the medical center, our sample of RNs from the three medical-surgical units generally reflects the sex and age profiles of RNs at our facility, and a similar percentage of Bachelor’s-prepared RNs. However, our sample contained more racial/ethnic diversity and more RNs with hospital diplomas (as opposed to Associate’s degrees). Finally, while facility-level data are only available with respect to years of federal service (as opposed to years of VA service specifically), it appears that nurses in our sample may have had fewer years with the VA system than RNs across the facility. This finding is not surprising as medical-surgical units have traditionally been settings in which new nursing graduates start their careers as generalists before moving on to more specialized settings such as intensive care.

However, at this time, our goal was not to produce generalizable knowledge applicable to all inpatient care settings, but to catalyze consideration and discussion of potential barriers and facilitators to implementation of inpatient screening, BI, and RT through exploration of these issues with front-line nurses engaged in direct care provision [72]. Because initiatives to improve the delivery of alcohol-related care within inpatient settings will most likely involve a high degree of nursing responsibility, we chose to focus on barriers and facilitators anticipated by the largest group of healthcare providers in general medical-surgical inpatient settings, i.e., nurses. Confirmation of these findings in additional studies, particularly in non-VA settings, is nonetheless warranted, as is inclusion of the perspectives of healthcare providers from other professional disciplines. We explored and identified *anticipated* barriers and *suggested* facilitators, instead of those already encountered by inpatient nurses at our facility because at this time, no local or national screening, BI, or RT implementation efforts are underway within VA inpatient settings.

## Conclusions

Nurse-delivered alcohol screening, BI, and potentially, RT, may be a novel approach to addressing risky alcohol use among hospitalized inpatients in the U.S. Despite anticipated patient-, provider-, and system-level barriers to implementation, nurse-delivered BI may constitute part of a viable model for BI delivery in the inpatient care setting after additional efficacy and effectiveness research is conducted. Calls have been issued for BI researchers to evaluate BI delivery models which are feasible at the pragmatic and economic levels [32]. Front-line healthcare providers can provide valuable perspectives informing the design, feasibility, and delivery of RCT interventions which can facilitate future translation of alcohol screening and BI into inpatient care delivery. Ongoing partnerships between health services researchers and nurses providing direct patient care in inpatient settings will facilitate the development, testing, and potential implementation of rigorous interventions designed to improve the identification, management, and prevention of unhealthy alcohol use in hospitalized patients.

## Competing interests

The authors declare that they have no competing interests.

## Authors’ contributions

LMB conceived of the study, participated in its design and coordination, performed data analysis/interpretation, and helped to draft, edit, and finalize the manuscript. KLR participated in the design of the study and focus group guide, moderated the focus groups, performed data analysis/interpretation, and helped to draft, edit, and finalize the manuscript. KLK helped to conceive the study, and helped to edit and finalize the manuscript. PAP assisted with data collection, analysis, and interpretation, and helped to edit and finalize the manuscript. MAS assisted with data interpretation and helped to edit and finalize the manuscript. AJG helped conceive the study, assisted in its coordination, and helped to edit and finalize the manuscript. All authors read and approved the final manuscript.

## Authors’ information

LMB is a Research Health Scientist at the Center for Health Equity Research and Promotion (CHERP), and the VISN 4 Mental Illness Research, Education, and Clinical Center (MIRECC) at the VA Pittsburgh Healthcare System in Pittsburgh, Pennsylvania, USA. She also serves as an Assistant Professor of Medicine at the University of Pittsburgh. Dr. Broyles is the recipient of a 5-year VA Career Development Award from the Health Services Research & Development service of the U.S. Department of Veterans Affairs. She is also the 2009 Council for the Advancement of Nursing Science/American Nurses Foundation (CANS/ANF) Scholar, and serves as at Member-at-Large on the Executive Committee of the Association for Medical Education and Research in Substance Abuse (AMERSA).

KLR is a Research Health Scientist at CHERP and the Veterans Engineering Resource Center (VERC) at the VA Pittsburgh Healthcare System. She is a founding member and Associate Director of the CHERP Qualitative Research Core. She also serves as an Assistant Professor of Medicine at the University of Pittsburgh, where she is affiliated with the Center for Bioethics and Health Law, the Institute for Doctor-Patient Communication, and the Center for Research on Health Care.

KLK is an Associate Professor of Medicine, Clinical & Translational Science, and Health Policy & Management at the University of Pittsburgh and Director of the RAND-University of Pittsburgh Scholars Program.

PAP is a staff nurse in the Critical Care Service Line at the VA Pittsburgh Healthcare System in Pittsburgh, Pennsylvania, USA, and a graduate student in the Family Nurse Practitioner Program at Duquesne University, also in Pittsburgh, PA.

MAS is a Research Health Scientist at CHERP and GRECC at the VA Pittsburgh Healthcare System. She is a Professor of Medicine, Public Health, and Clinical and Translational Science at the University of Pittsburgh.

AJG is a CHERP Core Investigator, Clinical Director of the VISN 4 MIRECC, and a staff physician at the VA Pittsburgh Healthcare System. He also serves as an Associate Professor and Advisory Dean at the University of Pittsburgh School of Medicine.
